# Quantitative three-dimensional myocardial perfusion cardiovascular magnetic resonance with accurate two-dimensional arterial input function assessment

**DOI:** 10.1186/s12968-015-0212-3

**Published:** 2015-12-04

**Authors:** Lukas Wissmann, Markus Niemann, Alexander Gotschy, Robert Manka, Sebastian Kozerke

**Affiliations:** Institute for Biomedical Engineering, University and ETH Zurich, Gloriastrasse 35, 8092 Zurich, Switzerland; Clinic of Cardiology, University Hospital Zurich, Zurich, Switzerland; Furtwangen University, Faculty Mechanical and Medical Engineering, Villingen-Schwenningen, Germany; Department of Internal Medicine, University Hospital Zurich, Zurich, Switzerland; Institute of Diagnostic and Interventional Radiology, University Hospital Zurich, Zurich, Switzerland; Imaging Sciences and Biomedical Engineering, King’s College London, London, UK

**Keywords:** DCE-MRI, First-pass myocardial perfusion imaging, Arterial input function, Myocardial blood flow estimates, Dual-sequence imaging

## Abstract

**Background:**

Quantification of myocardial perfusion from first-pass cardiovascular magnetic resonance (CMR) images at high contrast agent (CA) dose requires separate acquisition of blood pool and myocardial tissue enhancement. In this study, a dual-sequence approach interleaving 2D imaging of the arterial input function with high-resolution 3D imaging for myocardial perfusion assessment is presented and validated for low and high CA dose.

**Methods:**

A dual-sequence approach interleaving 2D imaging of the aortic root and 3D imaging of the whole left ventricle using highly accelerated *k-t* PCA was implemented. Rest perfusion imaging was performed in ten healthy volunteers after administration of a Gadolinium-based CA at low (0.025 mmol/kg b.w.) and high dose (0.1 mmol/kg b.w.). Arterial input functions extracted from the 2D and 3D images were analysed for both doses. Myocardial contrast-to-noise ratios (CNR) were compared across volunteers and doses. Variations of myocardial perfusion estimates between volunteers and across myocardial territories were studied.

**Results:**

High CA dose imaging resulted in strong non-linearity of the arterial input function in the 3D images at peak CA concentration, which was avoided when the input function was derived from the 2D images. Myocardial CNR was significantly increased at high dose compared to low dose, with a 2.6-fold mean CNR gain. Most robust myocardial blood flow estimation was achieved using the arterial input function extracted from the 2D image at high CA dose. In this case, myocardial blood flow estimates varied by 24 % between volunteers and by 20 % between myocardial territories when analysed on a per-volunteer basis.

**Conclusion:**

Interleaving 2D imaging for arterial input function assessment enables robust quantitative 3D myocardial perfusion imaging at high CA dose.

## Background

Qualitative assessment and quantitative evaluation of conventional first-pass perfusion cardiovascular magnetic resonance (CMR) is subject to a trade-off pertaining to contrast agent (CA) dosage. While high CA dose leads to satisfactory myocardial contrast enhancement, facilitating qualitative discrimination between ischemic and healthy myocardial tissue [1, 2], it results in pronounced non-linearity between signal intensity and CA concentration in the blood pool [3, 4]. Accordingly, the signal enhancement during peak bolus passage is reduced, resulting in underestimation of the arterial input function (AIF) and overestimation of myocardial blood flow (MBF) estimates [5]. The issue can be addressed by acquiring the AIF and the myocardial tissue signals with different time delays after saturation or inversion preparation [6, 7], or by using a dual-bolus approach [1, 8].

MBF estimation from first-pass contrast-enhanced CMR data is based on a linear time-invariant impulse response model [9]. Knowledge of both the AIF and the myocardial signal intensity-time curves is needed to calculate estimates of MBF per unit muscle mass [5]. Conventionally, both the AIF and the myocardial signal intensity-time curves are extracted from the same CMR image [5]. The application of low CA dose ensures an approximately linear relationship between signal intensity and CA concentration, hence facilitating a simple conversion from signal to concentration. Accordingly, low CA dose has been advocated for quantitative perfusion CMR. Higher CA dose, however, yields better CNR resulting in improved detectability of ischemic regions using qualitative assessment [10]. For a quantitative estimation of MBF, the AIF may be acquired separately using a low resolution image in conjunction with high-resolution image data to obtain myocardial signals [6].

Various approaches have been proposed to avoid non-linearity in the conversion from signal to concentration while exploiting the benefits of high CA dose. All methods proposed so far are based on either a reduction of the dose for the AIF acquisition or modification of the time between magnetization preparation and imaging, e.g. the saturation recovery time in case of saturation preparation. Dual-bolus approaches employ a low dose bolus for the AIF measurement in a separate scan followed by a higher dose to obtain the myocardial residue curves [1, 8, 11]. Dual-sequence methods use two interleaved imaging sequences to acquire the AIF and tissue residue concurrently from the same CA bolus [6, 7, 12]. An alternative sequence-based approach is the use of radial trajectories and reconstruction of separate images for blood pool and myocardial enhancement from different amounts of projections in the same dataset [13–15]. While the dual-bolus approach results in increased overall scan times and the administration of multiple CA injections adds to the complexity of the exam, examination time and CA setup are not changed in the dual-sequence approach. Accordingly, the dual-sequence approach is preferred and considered suited for wider clinical adoption.

In order to address the limited cardiac coverage of two-dimensional (2D) multi-slice myocardial perfusion CMR techniques, three-dimensional (3D) methods have been developed based on scan acceleration methodology [16–19]. Recent multi-centre data have confirmed the diagnostic accuracy of 3D CMR perfusion imaging [20, 21], and quantification of the percentage of ischemic myocardium by direct volumetry has been demonstrated [20, 22, 23]. Since ischemic burden above 10 % is increasingly used as a marker for decision making as to the need for revascularization [24], 3D CMR perfusion imaging is expected to play an important role as a technique for the diagnosis of stable coronary artery disease. The added value of deriving quantitative MBF estimates from CMR data has also been emphasized in the context of triple vessel coronary artery disease [25] and syndrome X [26], and the feasibility of whole-heart MBF estimation from 3D CMR perfusion images has recently been reported [27].

The present study introduces a dual-sequence approach interleaving 3D high-resolution myocardial perfusion imaging and 2D low-resolution AIF acquisition for high dose first-pass perfusion imaging with whole-heart coverage. Using in-vivo data obtained in healthy volunteers at rest it is demonstrated that the sequence allows for improved AIF assessment and hence improved MBF estimation.

## Methods

A dynamically interleaved 2D/3D dual-sequence scheme was implemented in a dedicated acquisition framework [28]. All images were acquired on a 1.5 T Philips Achieva MR system (Philips Healthcare, Best, The Netherlands) using a 5-element cardiac receive coil array. Interleaved acquisitions consisted of electrocardiogram (ECG) triggered saturation-recovery spoiled gradient echo sequences using spatiotemporal *k-t* undersampling. Gadobutrol (Gadovist, Bayer Schering Pharma, Germany) was used as contrast agent (CA).

### Phantom measurements

Saline phantoms doped with variable amounts of CA were built. CA concentrations varied from 0 to 5 mmol/l. Using the relationship between *T*_1_ and concentration *c*,

1 $$ \frac{1}{T_1}=\frac{1}{T_{1,0}}+c\cdot R $$

with CA relaxivity $R=5.2\mathrm{l}/\mathrm{mmol}\cdot \mathrm{s}$[29] and *T*_1,0_ = 1200 ms at baseline in the absence of CA [30], peak concentration corresponded to *T*_1_ = 37 ms. This value is in line with previous studies, where blood pool *T*_1_ values at peak bolus between 30 and 50 ms were reported [6, 12].

Phantoms were measured using saturation recovery delays of 30 and 150 ms to investigate the relationship between signal intensity and concentration. For the 2D-AIF sequence the signal to concentration linearity must be approximately valid in the blood pool at peak enhancement, hence a short saturation delay is required. For the 3D acquisition, the linearity must hold for the myocardial tissue only, where the expected *T*_1_ is above 200 ms [12], thus enabling longer saturation delays.

### In-vivo experiments

Ten healthy volunteers (5 male) with an average age (± standard deviation) of 25.7 ± 5.1 years underwent first-pass rest perfusion CMR examinations. All volunteers were scanned upon written informed consent according to local ethics regulations. Two contrast-enhanced dual-sequence imaging experiments were run using CA boluses at doses of 0.025 and 0.1 mmol/kg b.w. to compare low and high dose imaging. CA was injected at 4 ml/s and followed by a 30 ml saline flush at the same rate using a power injector (Medrad, Indianola, PA, USA). Twenty minutes were allowed for CA washout in-between the two bolus injections; low CA dose imaging was always performed first. Image acquisition covered 30 heartbeats during a single breath-hold.

**Fig. 1:**
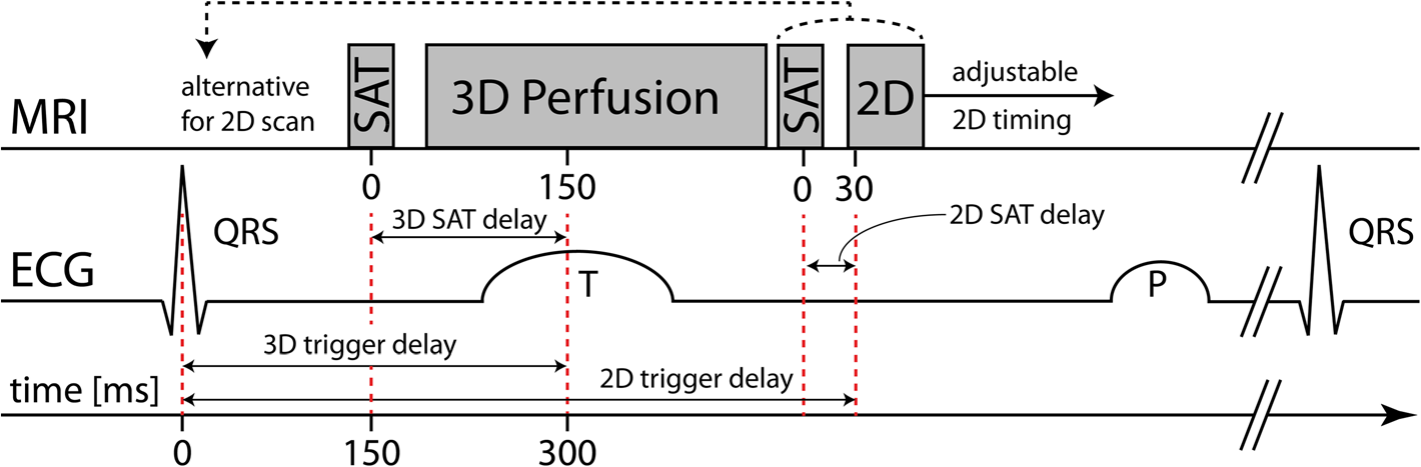
Dual-sequence diagram and corresponding ECG curve. The 3D perfusion scan is triggered to end systole with a saturation delay of 150 ms between saturation pulse (SAT) and *k-*space centre. The 2D-AIF images are acquired in the aorta during diastole with a 30 ms saturation delay. 2D image timing can be adapted to a diastolic time frame. Alternatively, for stress imaging, the 2D sequence can be run immediately after the R-peak before the 3D perfusion scan

**Fig. 2:**
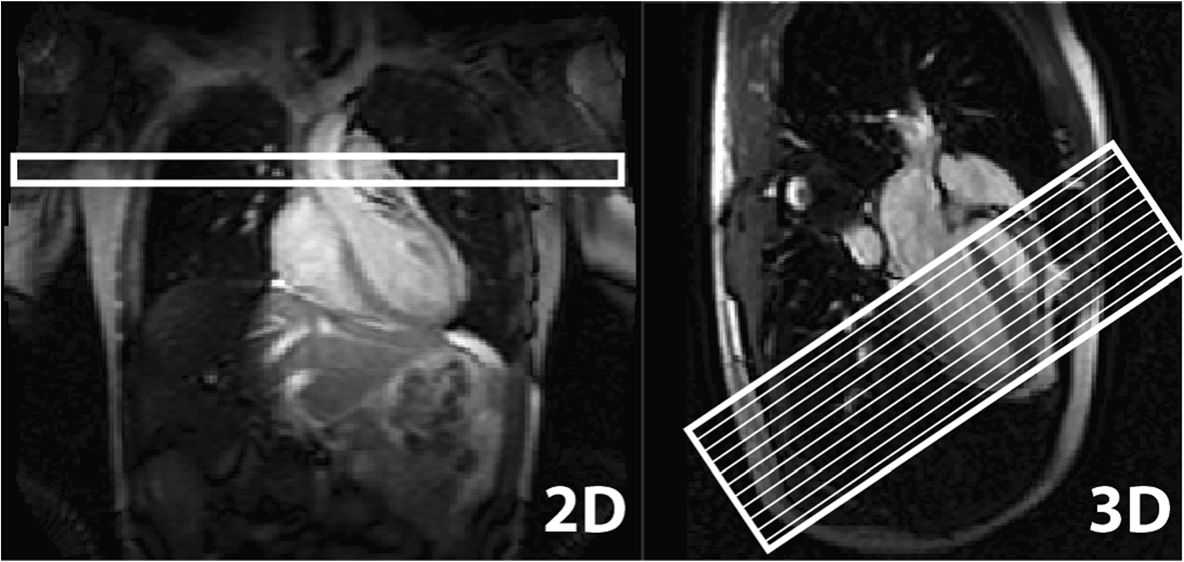
Typical 2D and 3D perfusion scan planning. The 2D-AIF acquisition was planned in the ascending aorta in transversal orientation. The survey scan was used as a basis and a stack of black-blood spin-echo images was included to determine the optimal slice location in feet-head direction. Short-axis 3D scans were planned based on a 3-chamber cine scan. 16 slices of 5 mm thickness from apex to base were reconstructed. Field-of-view and phase-encode directions were adjusted individually to avoid fold-over artefacts

Acquisition comprised ECG-triggered saturation-recovery spoiled gradient echo sequences with individual WET saturation preparation [31] played out in an interleaved fashion within each heartbeat, as shown in Fig. 1. 3D imaging was triggered to end systole and employed 10-fold Cartesian *k-t* undersampling [17, 32]. Ten contiguous short-axis slices were acquired using 75 % partial Fourier sampling in frequency-encode and both phase-encode directions. Elliptical *k-*space shutters were applied on the undersampling pattern as well as on the fully sampled 11x7 central *k-*space training matrix. The net undersampling factor was 7.1-8.2 depending on the field-of-view. Including partial Fourier sampling and *k-*space shutters, acceleration factors were between 12.2 and 15.2 when compared to a fully sampled Cartesian scan. 3D imaging parameters were: repetition time (*T*_*R*_): 2–2.2 ms, echo time (*T*_*E*_): 0.78-0.95 ms, spatial resolution: 2.0x2.0x10 mm^3^, flip angle: 15°, acquisition window: 226–309 ms, and saturation delay: 150 ms. Transverse 2D images were acquired in the ascending aorta. A diastolic time frame after aortic valve closure, identified using a 3-chamber cine scan, was selected for 2D imaging to avoid inflow effects [33]. Three-fold *k-t* undersampling with 11 training profiles was applied. 2D acquisition parameters were: spatial resolution: 3.5x3.5 mm^2^, slice thickness: 10 mm, flip angle: 15°, acquisition window: 56–64 ms, saturation delay: 30 ms, and 75 % partial Fourier sampling in *k*_*x*_ and *k*_*y*_. *T*_*R*_ and *T*_*E*_ were equal to the corresponding 3D image. Figure 2 illustrates typical scan planning for the interleaved 2D/3D measurement.

### Image reconstruction and signal comparison

Image reconstruction was performed using ReconFrame (Gyrotools LLC, Zurich, Switzerland). Subsequent post-processing was implemented in Matlab (The MathWorks, Natick, MA, USA). Image reconstruction was performed using *k-t* principal component analysis (*k-t* PCA) [34] with a separate sensitivity reference scan for coil sensitivity map calculation. Zero-filling was employed to achieve reconstruction resolutions of 2.0x2.0 mm^2^ and 1.25x1.25x5 mm^3^ for 2D and 3D images, respectively. The ascending aorta was segmented manually in the 2D image to obtain the corresponding arterial input function (2D-AIF). The AIF from the 3D image (3D-AIF) was extracted from the left-ventricular blood pool in a mid-ventricular slice. Non-linearity effects in the 3D-AIF relative to the 2D-AIF were visualized using signal intensity-time plots. To match the signal level in both AIFs for visualization, the 3D-AIF was scaled to the 2D-AIF by a constant factor. This scaling factor was computed as the ratio of mean signal intensities in the last 4 time frames of the 2D-AIF and the 3D-AIF [13–15]. Scaling was only applied for visualization purposes, but not for the signal to concentration conversion discussed below.

### Contrast-to-noise ratio analysis

The left-ventricular myocardium was manually segmented in eight slices of the 3D data for low and high dose. Voxel-wise signal intensity-time curves were extracted to compute contrast-to-noise ratio (CNR) maps at low and high dose. CNR was defined as [35]

2 $$ \mathrm{C}\mathrm{N}\mathrm{R}={\mathrm{S}\mathrm{N}\mathrm{R}}_{t, \max }-{\mathrm{S}\mathrm{N}\mathrm{R}}_{t,\mathrm{base}}=\frac{{\mathrm{S}}_{t, \max }-{\mathrm{S}}_{t,\mathrm{base}}}{\sigma_{\mathrm{base}}}. $$

In this equation, *S*_*t,*max_ and *S*_*t,*base_ are the signal intensities at time points of maximum contrast enhancement and baseline. The noise level *σ*_base_ is given by the standard deviation of the signal at baseline in all myocardial signal intensity-time curves. Example myocardial signal intensity-time curves and CNR maps were visualized and average whole-heart CNR values at low and high dose were compared.

### Signal intensity to concentration conversion

Average myocardial signal intensity-time curves were calculated in six circumferential sectors in eight slices of the 3D scan for myocardial blood flow (MBF) estimation. Signal intensity was converted to CA concentration using the signal model [9, 36]

3 $$ S={S}_0\left(\left(1- \exp \left(-{R}_1\cdot {T}_{\mathrm{SAT}}\right)\right)\cdot {a}^{n-1}+\left(1- \exp \left(-{R}_1\cdot {T}_R\right)\right)\cdot \frac{1-{a}^{n-1}}{1-a}\right), $$

with $a= \cos \alpha \cdot \exp \left(-{R}_1\cdot {T}_R\right)$. *T*_SAT_ is the saturation delay, *T*_*R*_ the repetition time, *α* the flip angle, *n* the number of profiles between acquisition start and *k-*space centre and *R*_1_ = 1/*T*_1_. *S*_0_ is a scaling factor proportional to the equilibrium magnetization. In a first step, *S*_0_ was estimated using the baseline signal and literature *T*_1_ values for the left-ventricular blood pool (1200 ms) and myocardial tissue (870 ms), respectively [30]. Using *S*_0_, which remains constant during the experiment, *T*_1_ was calculated for each time frame of the dynamic contrast-enhanced image. Finally, *T*_1_ values were inserted into equation (1) and the concentration *c(t)* was calculated for each time frame *t*.

### Myocardial blood flow quantification

The relationship between the concentration AIF *c*_AIF_(*t*) and the myocardial concentration-time curves *c*_MYO_(*t*) can be expressed by a convolution [37],

4 $$ {c}_{\mathrm{MYO}}(t)={R}_F(t)\otimes {c}_{\mathrm{AIF}}(t), $$

where

5 $$ {R}_F(t)=F\cdot R(t) $$

is the flow-weighted impulse response, *F* the estimate of MBF and *R*(*t*) a monotonically decaying function with *R*(*t*=0) = 1. MBF quantification was performed using Fermi model deconvolution [9], i.e. the impulse response in equation (4) was approximated by a Fermi function,

6 $$ {R}_F(t)=F\cdot \frac{1+\beta }{1+\beta \cdot {e}^{\alpha t}}, $$

with fitting parameters *α*, *β*, *F*. The quantification procedure comprised multiple steps. First, *c*_AIF_(*t*) was replaced by a gamma-variate function [38] to extract the first-pass AIF only [39]. Second, the temporal shift in bolus arrival time between the AIF and the myocardium was determined. The AIF was time-shifted by 0 to 6 s in steps of 0.5 s followed by Fermi deconvolution fitting. The temporal shift with smallest corresponding fitting error was then selected as the bolus arrival time shift, as suggested in [40]. The median of all regional time shifts in a dataset was used as a global time shift [40]. MBF quantification was performed for the low and high dose perfusion data using *c*_AIF_(*t*) from the 2D-AIF and the 3D-AIF, respectively.

### Statistical analysis

Statistical significance between low and high dose CNR results and estimated MBF using 2D- and 3D-AIFs was assessed on a per-subject basis (*N* = 10). The two-tailed paired Student’s *t*-test with *p* < 0.05 significance level was used for all evaluations. Bonferroni correction was applied for the statistical analysis of MBF estimates, where multiple *t*-tests of the same groups were performed.

## Results

**Fig. 3:**
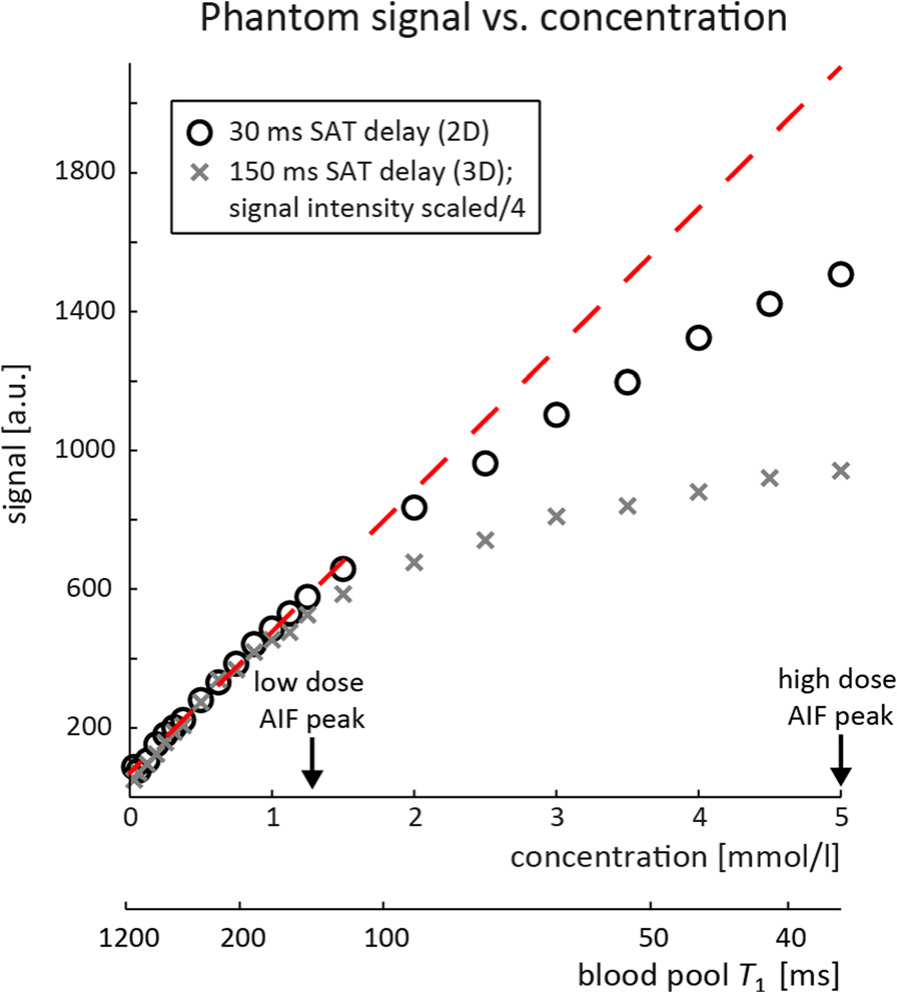
Measured phantom signal intensity vs. contrast agent concentration. Signal intensity vs. phantom CA concentration was measured for 2D and 3D saturation (SAT) delays used in-vivo. Corresponding *T*_1_ values calculated using equation (1) and baseline *T*_1_ = 1200 ms are shown alongside. Approximate peak concentrations at low and high dose are indicated by arrows. For low enough CA concentration, the linearity to the signal intensity is approximately valid (*dashed red line*). Non-linearity starts at concentrations of 1 and 2 mmol/l for saturation delays of 150 and 30 ms, respectively

The signal intensity for different concentrations of contrast agent in phantoms is plotted in Fig. 3. The saturation delays correspond to those used in-vivo for 2D (30 ms) and 3D imaging (150 ms). At low concentrations, signal intensity increases linearly with concentration. At higher concentrations linearity no longer holds, which is seen above 1 and 2 mmol/l contrast agent concentration for saturation delays of 150 and 30 ms, respectively.

**Fig. 4:**
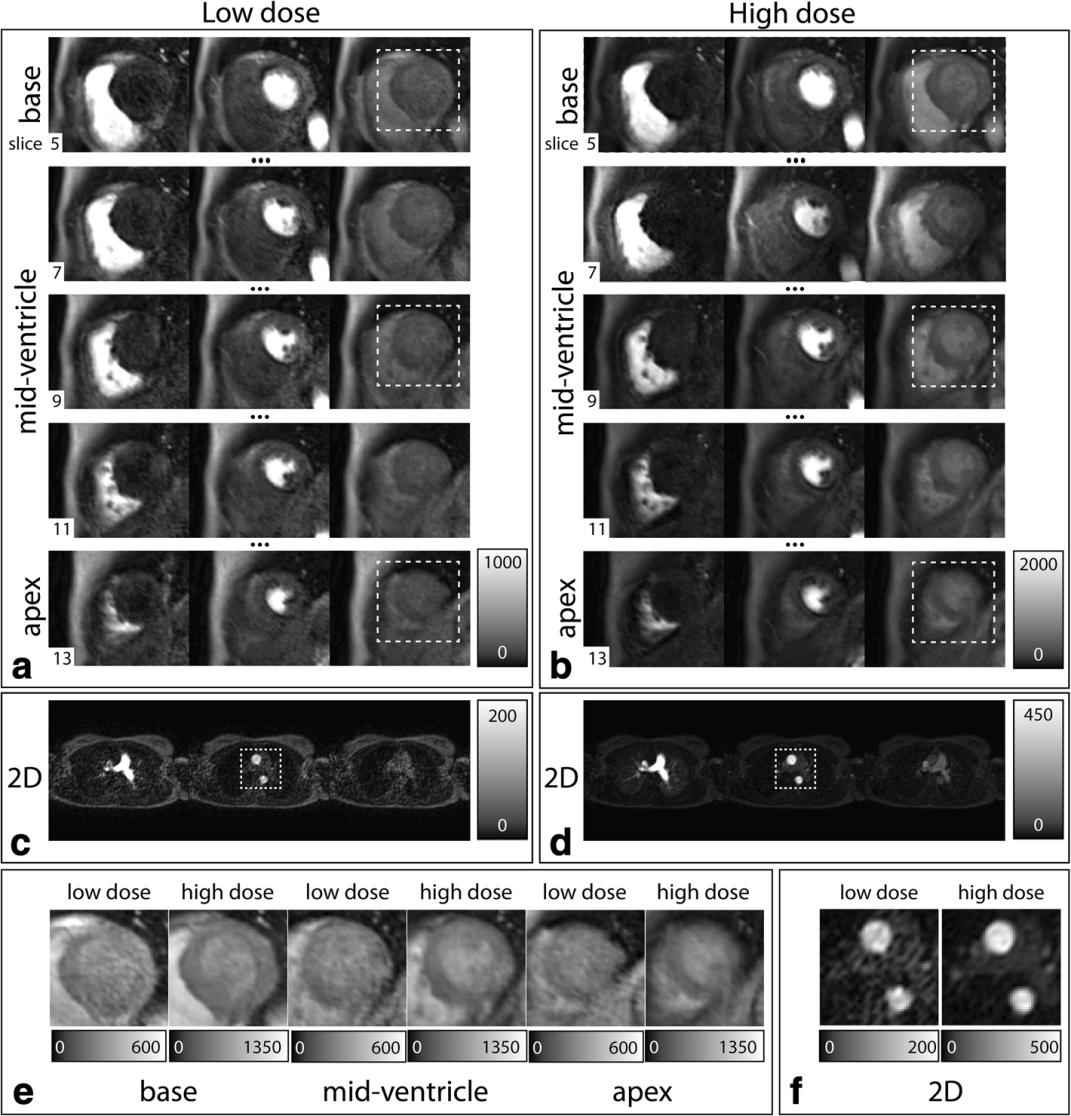
In-vivo 2D and 3D image examples at low and high dose. **a**, **b** Cardiac region of interest from five ventricular slices (apex to base) of the 3D images at peak contrast enhancement in the right ventricle, left ventricle and myocardium for low and high dose. **c**, **d** Low and high dose 2D images at the same time points. Grayscale values in low and high dose images are individually scaled to the peak values in the left ventricle (**a**, **b**) and ascending aorta (**c**, **d**), respectively. **e** Enlarged myocardial region of interest for 3 slices at peak myocardial enhancement, as indicated by the dashed boxes in (**a**, **b**). **f** Close-up of ascending and descending aorta at peak blood pool enhancement in the 2D-AIF images (dashed boxes in (**c**, **d**))

Example images of the 2D-AIF and 3D acquisitions are presented in Fig. 4 for peak contrast enhancement in the right ventricle, left ventricle and myocardium. Signal intensities in the 2D and 3D images are increased at high dose when compared to low dose data, as shown in Fig. 4e, f.

**Fig. 5:**
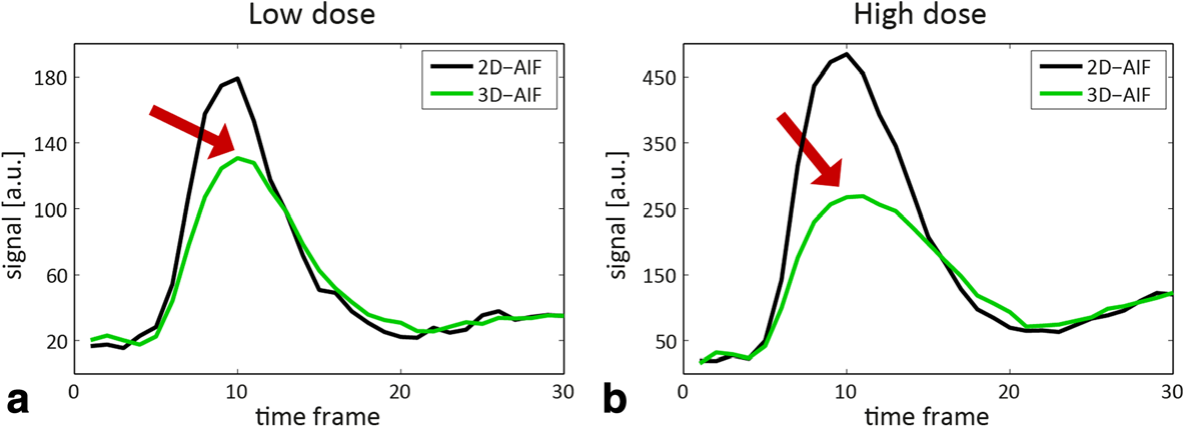
Comparison of in-vivo 2D-AIF and 3D-AIF at low and high dose. 2D-AIF vs. scaled 3D-AIF at (**a**) low and (**b**) high dose. The 3D-AIF was scaled by the average signal intensity ratio in the last four time points. Arrows highlight signal distortion of the 3D-AIF due to non-linearity between signal intensity and concentration

Figure 5 displays a comparison of in-vivo AIFs from one volunteer at low and high dose. Due to non-linearity between signal and concentration, the 3D-AIF shows a reduced signal enhancement during peak contrast bolus.

**Fig. 6:**
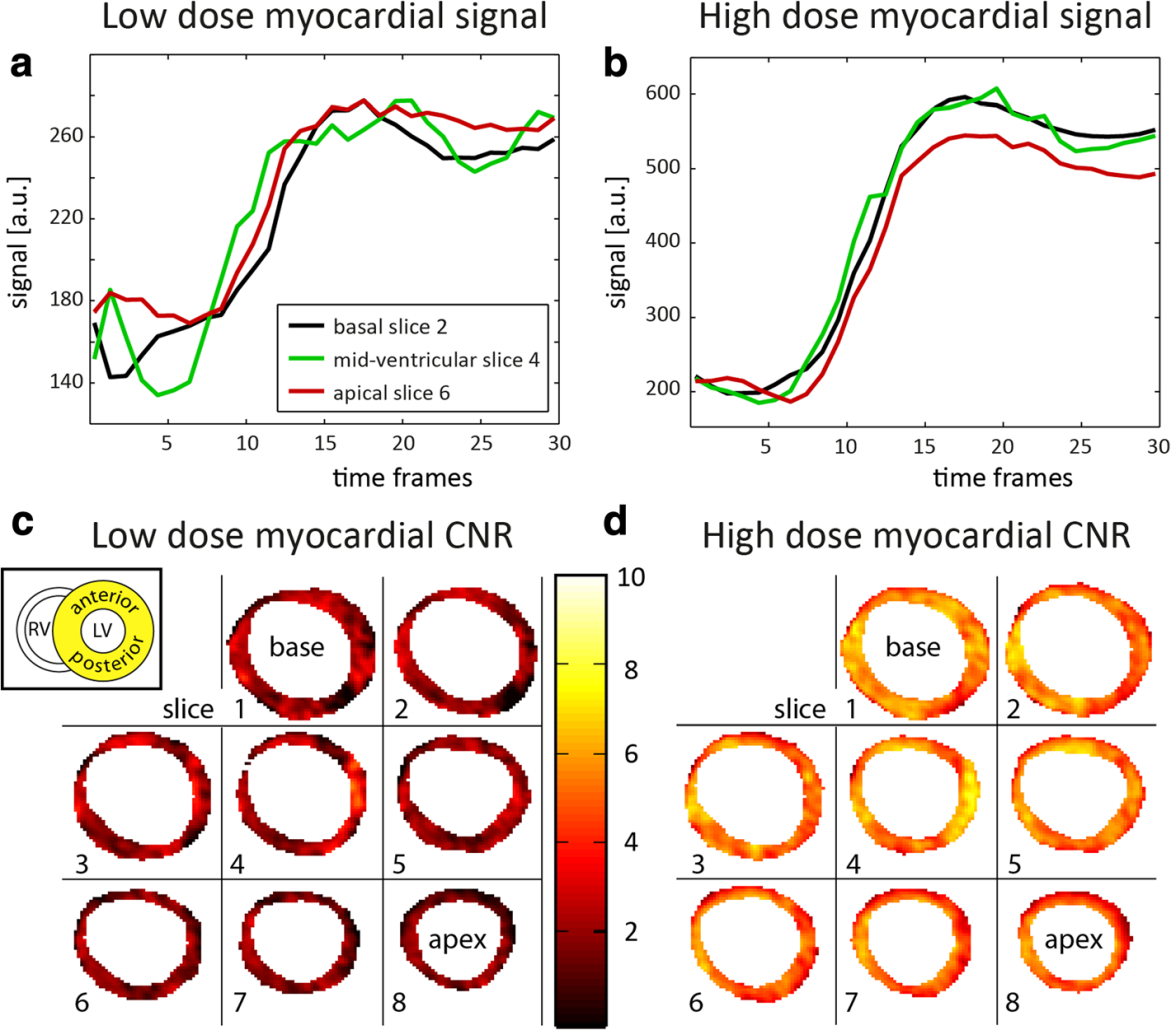
Myocardial signal intensity-time curves and CNR maps. **a**, **b** Example myocardial signal intensity vs. time curves at (**a**) low and (**b**) high dose. The curves show the mean signal from three different slices. **c**, **d** Voxel-wise myocardial contrast-to-noise ratio (CNR) maps for eight segmented slices between apex and base. CNR maps were oriented as indicated (inset). Slice numbers in (**a**, **b**) correspond to those in (**c**, **d**). High dose data shows more pronounced contrast enhancement and higher CNR values than low dose data. Mean CNR and standard deviations were 2.02 ± 0.88 and 5.23 ± 0.97 for low and high dose, respectively

Average myocardial signal intensity-time curves from three different slices and voxel-wise contrast-to-noise ratio (CNR) maps from eight slices are shown in Fig. 6. In this volunteer, mean CNR at high dose was 5.23 ± 0.97, while mean CNR at low dose was 2.02 ± 0.88.

**Fig. 7:**
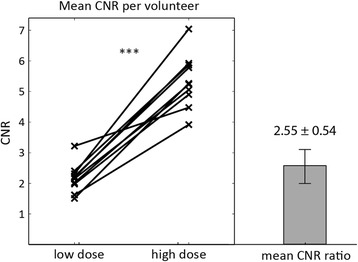
Average CNR per volunteer and CNR ratio. Mean myocardial CNR values at low and high dose for all volunteers. Data points corresponding to the same volunteer are connected. Mean CNR ratio and standard deviation across the study population are plotted on the same scale as CNR values. Statistical significance is indicated (*** = *p* < 0.001)

Mean CNR per volunteer and the overall CNR ratio are summarized in Fig. 7. Average CNR and standard deviation across volunteers was 5.34 ± 0.87 at high dose and 2.16 ± 0.47 at low dose. The average CNR was significantly higher at high dose compared to low dose (*p* < 0.001) with a mean CNR ratio of 2.55 ± 0.54.

**Fig. 8:**
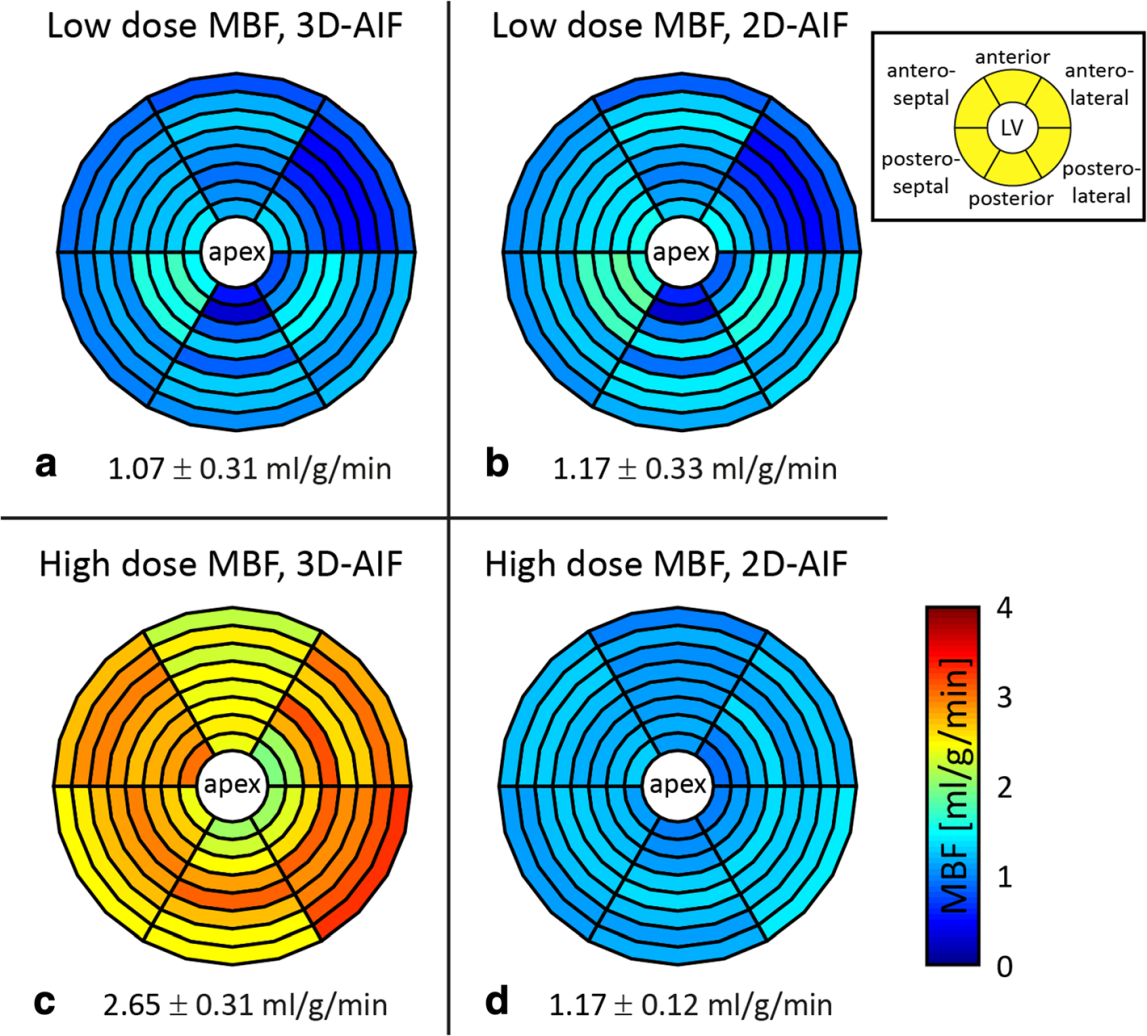
Example in-vivo myocardial blood flow (MBF) estimation results. Bull’s eye plots from low dose (**a**, **b**) and high dose (**c**, **d**) rest myocardial perfusion imaging in one volunteer. Eight slices were quantified using average myocardial curves in six angular sectors (inset). Quantification was performed using the 3D-AIF (**a**, **c**) and the 2D-AIF (**b**, **d**) as input functions. Numbers underneath indicate mean ± standard deviation of MBF estimates. Homogeneous MBF distribution across slices and angular segments is observed at high dose using the 2D-AIF

Example myocardial blood flow (MBF) estimation results are displayed in Fig. 8. The four subplots were generated by quantifying low and high dose data using both the 2D-AIF and the 3D-AIF. Bull’s eye plots indicate MBF estimates in eight slices on concentric circles from the most apical slice in the centre to the base outside. The myocardium was divided into six angular sectors yielding anterior, anterolateral, posterolateral, posterior, posteroseptal, and anteroseptal segments. Mean MBF estimates and standard deviations in this volunteer at low dose were 1.17 ± 0.33 and 1.07 ± 0.31 ml/g/min when quantified using the 2D-AIF and the 3D-AIF, respectively. Corresponding high dose values were 1.17 ± 0.12 and 2.65 ± 0.31 ml/g/min. Variation of MBF estimates at low dose was 29 % of the mean MBF estimate using both AIFs, while standard deviations at high dose were 12 % (3D-AIF) and 10 % (2D-AIF) for this volunteer. MBF estimates were more homogeneous across angular sectors and slices at high dose than at low dose.

**Fig. 9:**
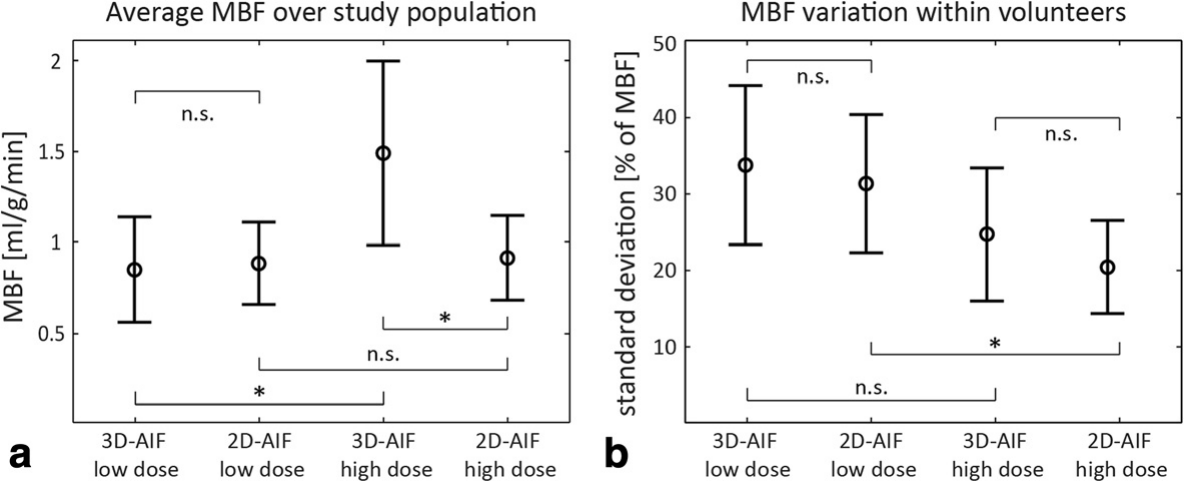
Myocardial blood flow quantification summary over all volunteers. **a** Mean and standard deviation of MBF estimates across the study population using the 2D-AIF and 3D-AIF for quantification at low and high dose. **b** Relative intra-volunteer mean and standard deviation of variation of MBF estimates for all four quantification datasets. Variation of MBF estimates was determined as the standard deviation of the 48 regional MBF estimates (8 slices, 6 sectors) within a volunteer, normalised by the mean MBF. Statistical significance is reported (n.s. = not significant; * = *p* < 0.05)

Figure 9 shows mean estimates of MBF and standard deviations across the study population and average intra-volunteer MBF variation using 3D-AIF and 2D-AIF at low and high dose. Mean low dose MBF estimates quantified using the 3D-AIF and 2D-AIF were 0.92 ± 0.29 and 0.95 ± 0.23 ml/g/min, respectively. Values at high dose were 1.57 ± 0.51 ml/g/min (3D-AIF) and 0.98 ± 0.24 ml/g/min (2D-AIF). Per-subject mean MBF estimates were significantly different between quantification using 2D-AIF and 3D-AIF at high dose, while there was no significant difference at low dose. When comparing low and high dose on a per-subject basis, no significant difference was found employing 2D-AIF-derived mean MBF estimates. On the other hand, there was a significant difference between low and high dose quantification using the 3D-AIF. Mean relative intra-volunteer variation of MBF estimates is presented in Fig. 9b. Relative variation for each volunteer was calculated as the standard deviation of the 48 regional MBF estimates normalised by the mean MBF value and expressed in percent. Mean variation in MBF estimates was 33.7 ± 10.4 % for the 3D-AIF and 31.3 ± 9.1 % for the 2D-AIF at low dose. Using the high dose data, intra-volunteer MBF variation reduced to 24.6 ± 8.7 % using the 3D-AIF and 20.3 ± 6.1 % with the 2D-AIF. Variation of MBF estimates was significantly different between low and high dose for the 2D-AIF, but not for the 3D-AIF. Moreover, the difference in MBF variation between 2D- and 3D-AIF for both doses was not significant.

## Discussion

In this study a dual-sequence approach for high-resolution quantitative 3D first-pass myocardial perfusion CMR has been presented. By using interleaved 2D and 3D acquisitions in each heartbeat, sequence timing for blood pool and tissue enhancement were separately adjustable. By comparing image sets acquired during high and low contrast agent dose administration, it was shown that high dose imaging yields increased CNR. Furthermore, if saturation delays are properly optimized, the proposed acquisition scheme allows for accurate measurement of the AIF. Non-linearity of the AIF around its peak is avoided if the saturation delay is sufficiently short such that the signal intensity remains proportional to contrast agent concentration.

The image acquisition framework employed for scan interleaving allowed for independent planning of the individual scans. A stack of short-axis slices covering the entire left ventricle was used for 3D imaging [20, 22]. The 2D-AIF scan was planned as a transverse slice outside the 3D volume through the ascending aorta [41]. This approach prevented additional saturation in the 3D imaging region and thus facilitated signal intensity to concentration conversion. By using equal flip angles, repetition and echo times for the 2D and 3D imaging modules, signal intensity differences mainly depended on different saturation delays. A similar approach using mid-ventricular 2D slice location [42] was rejected because of partial saturation of the 3D signal by the 2D sequence, yielding more complicated signal to concentration conversion. Furthermore, 2D imaging is more reliable than a pencil-beam probe acquisition [43], which is prone to inter-scan motion leading to partial volume effects.

A high contrast agent dose of 0.1 mmol/kg b.w. was compared to a low dose of a quarter of this amount. While the high dose 3D-AIF exhibited strong non-linearity effects, the quarter dose was just below the threshold of 0.03 mmol/kg suggested for MBF quantification with only minor non-linearity effects [5]. The small difference between 2D-AIF and 3D-AIF at low dose supports previous findings. Nevertheless, high dose 3D images had significantly higher CNR values than low dose images, with a 2.6-fold gain in CNR on average. Imaging experiments were performed on a 1.5 T MR system. However, the proposed protocol is readily applicable at 3 T, which is expected to further enhance CNR [44, 45].

Conversion of signal intensity to contrast agent concentration was performed using the signal model. In contrast to scaling of multiple AIFs to each other by a constant [6], signal model based conversion implicitly corrects for sequence parameter differences and the different dimensionality of the images by means of the scaling factor *S*_*0*_ in equation (3). Therefore, no normalization of the 2D to the 3D image is required. The signal model employed did not account for the efficiency of the WET saturation pulse train and thus perfect saturation was assumed. However, inefficient saturation might lead to additional errors in quantified MBF estimates, especially if saturation efficiency exceeds 1 [46]. MBF quantification was performed using Fermi model deconvolution, since it is a widespread and well-accepted model for first-pass perfusion analysis [47]. Furthermore, comparisons with other quantification methods revealed that Fermi deconvolution is more robust to noise [48, 49] and not inferior to other methods [50].

Estimates of MBF were computed using the 2D-AIF and the 3D-AIF as blood pool inputs, respectively. The small myocardial CNR at low dose caused large intra-volunteer variation of mean MBF estimates using both AIFs. At high dose, quantification using the 3D-AIF data yielded lower intra-volunteer, but even larger inter-volunteer variation of perfusion estimates. In this case, non-linearity effects compromised signal intensity to concentration conversion of the AIF, which resulted in global MBF estimation offsets. On the other hand, the superior myocardial CNR reduced variations between myocardial regions. Quantification using the 2D-AIF at high dose yielded smallest intra-subject variations of MBF estimates.

In order to compare low and high dose imaging, equal sequence parameters were chosen for both low and high contrast agent dose in this study. In a clinical setting, however, the dose is usually fixed and the sequence parameters are optimized accordingly. Given the low noise level in the 2D-AIF at high dose in all volunteers, the optimal saturation delay for 2D imaging at a contrast agent dose of 0.1 mmol/kg b.w. is below the 30 ms chosen for this study. In principle, the saturation delay can be arbitrarily short as long as the blood pool CNR is high enough. If the 2D sequence is run with a very short saturation delay, it can be acquired right after the R-peak before onset of systolic flow in the aorta. This was not possible in this study, since the saturation delay of 30 ms shifts the 2D acquisition in time and introduces a risk of contamination of the signal by the large inflow effects in systole [33].

Short examination times and applicability during pharmacological stress are key criteria for clinical feasibility of a myocardial perfusion CMR protocol. In contrast to dual-bolus imaging, dual-sequence methods enable acquisition of multiple images after a single bolus of contrast agent. This makes the proposed acquisition scheme compatible with established clinical protocols in terms of examination time. The temporal resolution was one 2D/3D image pair per heartbeat. Since heart rates can significantly increase during stress, acquisition during systole and early diastole is advisable. 3D imaging was triggered to end systole, where the myocardium was contracted and relatively quiescent. In addition to the thicker myocardial wall compared to diastolic imaging, measurement in systole is also favourable in terms of image quality and artefacts [27]. 2D images were acquired in diastole after aortic valve closure to suppress inflow effects [33]. On average over all volunteers, 2D/3D acquisition ended 700 ms after the R-peak enabling heart rates of up to 86 min^−1^. At the higher heart rates as expected during stress, and with the shortened 2D saturation delay recommended at high dose, 2D acquisition can be performed directly after the R-peak. At the same time the 3D acquisition window needs to be reduced to avoid motion induced image artefacts by shortening TR. While relatively long TRs of 2–2.2 ms were chosen in the present study, optimization of sequences timing allows TRs of 1.8–1.9 ms, hence enabling acquisition windows of 215–270 ms for 2x2x10 mm^3^ resolution depending on the field-of-view. For clinical stress imaging, it may be necessary to trade some spatial resolution to reduce the acquisition window further, as demonstrated previously for 2.3x2.3x10 mm^3^ voxel size [17, 21]. With sequence modifications as described above, and assuming an end-systolic trigger delay of 300 ms as well as 2/3 of the profiles acquired after that trigger delay, acquisition ends earlier than 500 ms after the R-peak. Accordingly and with the proposed modifications, the sequence is compatible with heart rates of at least 120 min^−1^. Furthermore, the saturation delay for 3D imaging is also freely adjustable, which again relaxes timing constraints.

## Conclusions

Interleaving 2D imaging for arterial input function assessment enables improved 3D myocardial perfusion imaging at high contrast agent dose. Short magnetization preparation times in 2D-AIF imaging allow for accurate input function sampling with only minor signal to concentration non-linearity. 3D CMR timing can be optimized to deliver high contrast-to-noise ratio regardless of non-linearity in the blood pool, yielding increased myocardial contrast and more robust myocardial blood flow estimates.

## Ethics, consent and permissions

This study was approved by the Ethics Committee of the Canton of Zurich (KEK) under the reference number EK-1294. All volunteers gave written informed consent for participation in this study. Consent to publish data from individual volunteers was obtained from all participants.

## Abbreviations

AIF: Arterial input function
CA: Contrast agent
CMR: Cardiovascular magnetic resonance
CNR: Contrast-to-noise ratio
ECG: Electrocardiogram
MBF: Myocardial blood flow
PCA: Principal component analysis
